# Human vascular adhesion proteın-1 (VAP-1): Serum levels for hepatocellular carcinoma in non-alcoholic and alcoholic fatty liver disease

**DOI:** 10.1186/1477-7819-8-83

**Published:** 2010-09-17

**Authors:** Ozgur Kemik, Aziz Sümer, Ahu Sarbay Kemik, Veyis İtik, Ahmet Cumhur Dulger, Sevim Purisa, Sefa Tuzun

**Affiliations:** 1 Department of General Surgery, Yuzuncu Yıl University Medical Faculty, Van, Turkey; 2 Department of Biochemistry, Cerrahpasa Medical Faculty, University of Istanbul, Istanbul, Turkey; 3 Department of Gastroeneterology, Yuzuncu Yıl University Medical Faculty, Van, Turkey; 4 Department of Biostatistics, Istanbul Medical Faculty, University of Istanbul, Istanbul, Turkey; 5 II. General Surgery, Haseki Research and Education Hospital, Istanbul, Turkey

## Abstract

**Background:**

The incidence of hepatocellular cancer in complicated alcoholic and non-alcoholic fatty liver diseases is on the rise in western countries as well in our country. Vascular adhesion protein-1 (VAP-1) levels have been presented as new marker. In our study protocol, we assessed the value of this serum protein, as a newly postulant biomarker for hepatocellular cancer in patients with a history of alcoholic and non-alcoholic fatty liver diseases.

**Methods:**

Pre-operative serum samples from 55 patients with hepatocellular cancer with a history of alcoholic and non-alcoholic fatty liver diseases and patients with cirrhosis were assessed by a quantitative sandwich ELISA using anti-VAP-1 mAbs. This technique is used to determine the levels of soluble VAP-1 (sVAP-1) in the serum.

**Results:**

sVAP-1 levels were evaluated in patients with hepatocellular cancer and liver cirrhosis. There was a significant difference in mean VAP-1 levels between groups. Serum VAP-1 levels were found higher in patients with hepatocellular cancer.

**Conclusion:**

These findings indicate that the serum level of sVAP-1 might be a beneficial marker of disease activity in chronic liver diseases.

## Introduction

Hepatocellular carcinoma (HCC) is a major health problem worldwide, with more than 5,00,000 cases diagnosed annually [1]. There has been an increase in the incidence of HCC over the last 5-8 years, however, the survival of those who have been diagnosed as HCC has not changed significantly in the last two decades [1,2].

Etiologies of the tumors in our HCC patients were mainly in alcoholic and non-alcoholic fatty liver diseases. Vascular adhesion protein-1 (VAP-1) has been used for the diagnosis of HCC arising from steatohepatitis associated with cirrhosis as an important marker.

VAP-1 is one of the endothelial cell adhesion molecules that mediate binding of lymphocytes to the endothelium under some conditions [3,4]. It is primarily expressed in high endothelial venules in peripheral lymph nodes. Furthermore, the expression of VAP-1 is induced and up-regulated by chronic inflammation in the vessels of the tonsil, gut, skin, and synovium [5]. VAP-1 is present on sinusoidal and vascular endothelia in the liver under both normal and inflammatory conditions [6].

The ripe VAP-1 molecule is a 170-kDa homodimeric glycoprotein that consists of two 90 kDa subunits connected together by disulfide bonds [7]. VAP-1 has a big extracellular domain, a single-pass trans-membrane domain, and a short cytoplasmic line [8].

The molecule has effusive sialic acid decorations that are descended to its tenacious function, but VAP-1 is incapable to mediate lymphocyte adhesion to desialylated vessels [7].

Recently, most of the leukocyte-endothelial cell adhesion molecules, such as ICAM-1, VCAM-1, platelet endothelial cell adhesion molecule-1, selectins (E-selectin, P-selectin, L-selectin) have been demonstrated to circulate in soluble forms [9-12]. Extracellular parts of L- and E-selectin, VCAM-1, and ICAM-1 are enzymatically ruptured from the cell surface and released into the bloodstream [13]. This process is one of the mechanisms by which soluble forms of adhesion molecules can be produced. It may be different from soluble adhesion molecules and contrasting physiologic effects. They may function as inhibitors of cell to cell adhesion by competing with their membrane-bound forms [14]. Alternatively, soluble molecules can trigger responses in cells that bear their ligands [15]. Moreover, because the levels of these adhesion molecules change in different states, the determination of their concentrations can be beneficial for the diagnosis and treatment and monitoring of diseases [9,12,16,17].

In this study, we aimed to demonstrate that sVAP-1 levels can be elevated in patients with alcoholic and non-alcoholic fatty liver diseases preceding hepatocellular cancer. We suggest that the increased levels of sVAP-1 may be a clinically useful marker in liver diseases.

## Materials and methods

History and clinical properties were interrogated and serum samples were collected after approval by Haseki Education and Research Hospital Ethics Committee approved this study. Informed consent was obtained from all patients. All patients were diagnosed with HCC as proposed by the European Association for the Study of the liver [18]. Pre-operative 55 patients with HCC, all of whom had cirrhosis, were selected for study. Of these, 33 patients had alcoholic liver disease and 22 patients non-alcoholic fatty liver disease. The diagnosis of non-alcoholic fatty liver disease cirrhosis was made in patients who had clinical features and liver biopsies compatible with non-alcoholic fatty disease. Females and males consuming greater than 15 or 20 units of alcohol per week respectively were excepted from this category, as were any individuals with viral or autoimmune liver diseases. The presence of steatosis and stage 4 fibrosis defined by modified Brunt criteria was necessary for the diagnosis [19]. Patients with positive results for either HBsAg or HCV were excluded from this study. Blood was transfered into serum tubes. After centrifugation at 3000 rpm for 10 min, serum was collected, aliquoted, and kept frozen at -70 °C, sVAP-1 was studied in this serum by ELISA.

### sVAP-1 ELISA

Wells of microtiter plates (96-well, flat-bottom, white Cliniplate EB; Labsystems) were coated with 100 μL of the anti-VAP-1 mAb TK8-18 at 10 μg/mL in 0.1 M NaHCO3 buffer (pH 9.6) at 4° C overnight and then at 37° C for 1 h. The wells were washed six times with 0.1% Tween 20 in PBS (Tween/PBS) and then blocked to prevent non-specific adsorption by the addition of 200 μL of PBS containing 1% gelatin and 1% nonfat milk powder, a blocking solution, for 45 min at room temperature. After washing the wells six times with Tween/PBS, 175 μL of each serum sample at 1:25 dilution in the blocking solution, it was added into the wells, and the plates were left at room temperature for 1 h. The wells were then washed six times with Tween/PBS and incubated with 100 μL of the biotinylated anti-VAP-1 mAb TK8-14 or biotinylated control mAB Hermes-3, 10 μg/mL in the blocking solution, at room temperature for 1 h. After washing six times with Tween/PBS, 100 μL of streptavidin-horse-radish peroxidase was added into the wells, and the plates were allowed to incubate at room temperature again for 1 h. Finally, plates were washed six times with Tween/PBS and generated after combining with chemiluminescence ELISA reagent (Boehringer Mannheim, Germany) according to the manufacturer's instructions. The intensities of the chemiluminescence reactions in the wells were always measured after a 3-min incubation time.

Each sample was measured in triplicate with both the anti-VAP-1 mAb and the negative control mAb. The specific VAP-1 value was calculated by subtracting the mean background value of the negative control from the mean value of VAP-1 [20].

### Statistical analysis

Data obtained by Shapiro Wilk normality test were done with leaf and steam. Correlations were performed using Spearman's correlation test.

## Results and discussion

Serum VAP-1 levels were determined in 55 patients with HCC arising from both alcoholic cirrhosis and non-alcoholic fatty liver disease. A control group consists of 46 patients with alcoholic cirrhosis and non-alcoholic fatty liver disease. The clinical characteristics of the patients in these groups are shown in Table 1.

**Table 1:** Clinical characteristics of the patients with cirrhosis and cirrhosis plus HCC.

	Cirrhosis	HCC
Number	46	55
Age (years)	56.4 ± 8.6	58.9 ± 7.3
Male:Female	27:19	39:16
Alcoholic:non-alcoholic	30:16	30:25
Portal vein invasion	NA	11
Single nodule	NA	14
Two nodules	NA	10
≥ 3 nodules	NA	18

NA: Not applicable.

Mean VAP-1 levels were significantly different between patients with HCC and liver cirrhosis, as shown in Table 2 (p < 0.01).

**Table 2:** Levels of VAP-1 (ng/mL).

	Cirrhosis	Total HCC	HCC-AFL	HCC-NAFL	p
	(n = 46)	(n = 55)	(n = 33)	(n = 22)	
VAP-1	98.4 ± 12.6	105.0 ± 10.2	104.3 ± 7.5	99.1 ± 3.9	< 0.01

HCC-AFL: HCC-associated with alcoholic liver diseases.

HCC-NAFL: HCC-associated with non-alcoholic liver diseases.

There were no age or gender differences between cirrhosis and HCC patient groups. However, serum VAP-1 levels were found higher in HCC than cirrhosis group (respectively; 98.4 ± 12.6, 105.0 ± 10.2) (p < 0.01).

There was a difference between levels of VAP-1 in patients with HCC- associated alcoholic liver diseases and with HCC-associated non-alcoholic liver diseases. We found VAP-1 on a significantly higher level in patients with HCC-associated alcoholic liver diseases (p < 0.01). But, there were correlation between levels of sVAP-1 in patients with HCC-associated alcoholic liver diseases and in patients with HCC-associated non-alcoholic liver diseases (p < 0.01, r = 0.61).

We found a correlation between the patients with total HCC and the patients with HCC-associated alcoholic liver diseases and HCC-associated non-alcoholic liver diseases (p < 0.01, r = 0.51). Also, there were correlation between healthy individuals and patients with total HCC (r = 0.59, p < 0.01).

The elevation incidence of HCC [2], is usually related to the the fact that the generality of these tumors are diagnosed at stage III or stage IV, when curative treatments are not possible [21].

Our results represent a tremendous amount of VAP-1. In our study, serum VAP-1 has a moderate significance as a biomarker of HCC in alcoholic cirrhosis and non-alcoholic fatty liver disease patients, with a sensitivity of 98% (10 ng/mL) in combination with a specificity of 100%. The threshold level of VAP-1 for diagnosis of HCC is a 100 ng/mL [20]. The concentrations of VAP-1 measured by ELISA in healthy person were found to be between 21 and 89 ng/mL [20]. No age or sex correlations were observed.

Significantly increased levels of VAP-1 were found in patients with HCC. Patients with HCC had the most marked elevations in systemic circulation. The increased serum levels in these patients were quite enough to suggest that the concentrations of VAP-1 could cause biologic effects. This finding is involved, because these patients are diagnosed and treatment as HCC.

In our study, we found higher levels of VAP-1 in patients with HCC-associated alcoholic liver diseases. This protein is significantly increased in the serum of HCC patients, but it is not elevated in neither HCV associated HCC patients nor patients with steatohepatitis related HCC, however patients diagnosed as steatohepatitis cirrhosis alone have high concentrations. The mechanism of this differentiation is unclear. It is possible that this newly protein as a candidate biomarker, mixed to other endothelial adhesion molecules, may yet improve their mechanisms.

Toiyama and collegaous [22] assessed the sVAP-1 levels in patients with colorectal cancer and they found a decrease in the sVAP-1 levels.

In conclusion, we have shown that sVAP-1 is present in the serum of patients with HCC. Moreover, the level of VAP-1 is increased in liver diseases in a more disease-specific manner than the levels of other known present adhesion molecules [16]. VAP-1 may dominant its biologic function by the endothelial cell adhesion cascade. We suggested that this protein may be a significant marker for HCC in patient with alcoholic and non-alcoholic fatty liver diseases.

## Competing interests

The authors declare that they have no competing interests.

## Authors' contributions

**OK, ST**- Collected data and wrote the manuscript in draft. **ASK**- Carried out the biochemical analysis. **SP**-Carried out the statistical analysis. **AS, ACD,VI**- Took part in and contributed the discussion. All authors have read and approve of the final manuscript.
